# *In vivo* RNA sequencing reveals a crucial role of Fus3-Kss1 MAPK pathway in *Candida glabrata* pathogenicity

**DOI:** 10.1128/msphere.00715-24

**Published:** 2024-10-30

**Authors:** Xinreng Mo, Xiangtai Yu, Hao Cui, Kang Xiong, Shan Yang, Chang Su, Yang Lu

**Affiliations:** 1 Hubei Key Laboratory of Cell Homeostasis, College of Life Sciences, Wuhan University, Wuhan, China; 2 Hubei Key Laboratory of Cell Homeostasis, College of Life Sciences, TaiKang Center for Life and Medical Sciences, Wuhan University, Wuhan, China; University of Georgia, Athens, Georgia, USA

**Keywords:** *Candida glabrata*, MAPK signaling pathway, virulence, *FUS3*, *KSS1*

## Abstract

*Candida glabrata* is an important and increasingly common pathogen of humans, particularly in immunocompromised hosts. Despite this, little is known about how this fungus causes disease. Here, we applied RNA sequencing and an *in vivo* invasive infection model to identify the attributes that allow this organism to infect hosts. Fungal transcriptomes show a dramatic increase in the expression of Fus3 and Kss1, two mitogen-activated protein kinases (MAPKs), during invasive infection. We further demonstrate that they are both highly induced under a combination of serum and high CO_2_ conditions. Deletion of both *FUS3* and *KSS1*, but neither gene alone, results in a reduced fungal burden in organs, as well as in the gastrointestinal tract in the DSS (Dextran Sulfate Sodium)-induced colitis model. Similarly, the defect in persistence in macrophages and attenuated adhesion to epithelial cells are observed when *FUS3* and *KSS1* are both disrupted. The *fus3 kss1* double mutant also displays defects in the induction of virulence attributes such as genes required for iron acquisition and adhesion and in the anti-fungal drug tolerance. The putative downstream transcription factors Ste12 (1), Ste12 (2), Tec1, and Tec2 are found to be involved in the regulation of these virulence attributes. Collectively, our study indicates that an evolutionary conserved MAPK pathway, which regulates mating and filamentous growth in *Saccharomyces cerevisiae*, is critical for *C. glabrata* pathogenicity.

**IMPORTANCE:**

The MAPK signaling pathway, mediated by closely related kinases Fus3 and Kss1, is crucial for controlling mating and filamentous growth in *Saccharomyces cerevisiae*, but this pathway does not significantly impact hyphal development and pathogenicity in *Candida albicans*, a commensal-pathogenic fungus of humans. Furthermore, deletion of Cpk1, the ortholog of Fus3 in pathogenic fungus *Cryptococcus neoformans*, has no effect on virulence. Here, we demonstrate that the MAPK pathway is crucial for the pathogenicity of *Candida glabrata*, a fungus that causes approximately one-third of cases of hematogenously disseminated candidiasis in the United States. This pathway regulates multiple virulence attributes including the induction of iron acquisition genes and adhesins, as well as persistence in macrophages and organs. Our work provides insights into *C. glabrata* pathogenesis and highlights an example in which regulatory rewiring of a conserved pathway confers a virulent phenotype in a pathogen.

## INTRODUCTION

*Candida glabrata* (*Nakaseomyces glabratus*), commonly residing in the oral and gastrointestinal regions of healthy humans, serves as a prevalent commensal organism (1). Nonetheless, it possesses the potential to transform into a pathogenic yeast, causing a range of infections from superficial to life-threatening systemic ones, with high morbidity and mortality rates. Factors such as weakened immune systems, cancer, the use of antibiotics, and the presence of intravenous catheters can increase the risk of contracting infections caused by *C. glabrata* (2). Among the emerging fungal pathogens, the prevalence of *C. glabrata* infections is on the rise, positioning it as the second most common source of candidiasis, ranking only behind *Candida albicans* (3). The challenge of treating infections caused by *C. glabrata* is increased by its natural resistance to antifungal treatments, a situation further complicated by its ability to adapt to azole-based fungistatic medications (4, 5).

Despite the rising incidence of *C. glabrata* infections, the molecular mechanisms underlying its virulence remain poorly understood. Unlike *C. albicans*, *C. glabrata* does not rely on a reversible morphogenetic switch between yeast and hyphal or pseudohyphal forms for pathogenicity. Although *C. glabrata* can switch to a pseudohyphal form under nitrogen-starvation conditions *in vitro* (6, 7), this filamentation process has not been observed under the *in vivo* conditions (8). Instead, the ability of *C. glabrata* to adhere strongly to various substrates has been found to be a key virulence attribute (9), which is mediated by a large number of adhesins. These adhesins are glycosylphosphatidylinositol (GPI)-modified proteins covalently incorporated into the cell wall (10) and play a critical role in the initial stages of infection, serving as the primary point of contact with the host. They are also crucial for biofilm formation on abiotic substrates, particularly on medical devices like catheters (11). The most well-studied adhesin family in *C. glabrata* is the Epithelial Adhesin (EPA) family, comprising approximately 17–23 genes depending on the isolate, which facilitates attachment to epithelial cells and macrophages (12–15). Among these EPA family adhesins, Epa1 is predominantly responsible for *in vitro* adherence to epithelial cells, as evidenced by a 95% reduction in adherence in *epa1* mutant cells (12). The YPS gene cluster, encoding extracellular GPI-linked aspartyl proteinases known as yapsins, is implicated in the removal and release of Epa1 (16). However, deletion of *EPA1* alone results in only a minor, non-significant reduction in organ colonization in a murine urinary tract infection model, likely due to the involvement of other adhesins in *C. glabrata* infection (17–19). Indeed, a triple mutant strain lacking *EPA1*, *EPA6*, and *EPA7* exhibits significantly reduced bladder colonization in this model (17).

In the model yeast *Saccharomyces cerevisiae*, adhesion and biofilm formation are mediated by the GPI-anchored cell wall protein *FLO11*, whose expression is regulated by the MAPK pathway and the cAMP/PKA pathway (20). The regulation via the MAPK pathway requires the transcription factors Ste12 and Tec1, whereas cAMP-mediated activation requires a distinct factor, Flo8 (21). One of the *STE12* homologs in *C. glabrata*, *STE12 (1*) has been found to be necessary for filamentation under the nitrogen-starvation condition and maintaining wild-type virulence in a murine model of candidiasis (22). A recent study reported a stronger biofilm-impaired phenotype, resulting from the loss of both *STE12*-homologous genes in the double mutant, compared with the single mutant with the deletion of *STE12*(1) (23). However, *C. albicans* mutant strain with the deletion of *CPH1*, the ortholog of *S. cerevisiae STE12*, is still able to cause lethal infections in mice (24). Instead, Flo8 functions downstream of the cAMP/PKA pathway, and together with a basic helix-loop-helix protein Efg1, are essential for the virulence of *C. albicans* (25). The mechanism of how the interconnections are established between downstream transcription factors of MAPK pathway and the *C. glabrata* pathogenicity needs to be further investigated.

MAP Kinases Fus3 and Kss1 play an essential role in maintaining the specificity of MAPK signaling in *S. cerevisiae* (26). These closely related kinases are activated by the common upstream MAPK kinase Ste7 yet generate distinct output responses, mating, and filamentous growth in *S. cerevisiae*, respectively (27). For the important human pathogenic fungus *Cryptococcus neoformans*, deletion of the *S. cerevisiae* Fus3 ortholog Cpk1 results in defects in mating pheromone production, cell fusion, and filamentous growth but does not affect virulence (28). Although it has been reported that deleting the *S. cerevisiae* Fus3 ortholog Cek1 in *C. albicans* led to a virulence defect in a mouse model of systemic infection (29), this finding may be confounded by the effect of the selectable marker gene (30). In the *C. glabrata* genome, homologs of *S. cerevisiae FUS3* and *KSS1* are present, but so far, they are not characterized.

Here, we found that the expression of MAPKs Fus3 and Kss1 was activated over the course of invasive infection of *C. glabrata*. Importantly, deletion of both *FUS3* and *KSS1* led to a reduced colonization in murine models of systemic candidiasis and DSS-induced colitis. Moreover, we demonstrate the critical role of *C. glabrata* MAPKs Fus3 and Kss1 in the resistance to intracellular killing by macrophages and adhesion to the plastic surface and human epithelial cells. The downstream transcription factors of MAPK signaling pathway have also been identified to be involved in the regulation of *C. glabrata* virulence. Our findings suggest that the activation of MAPK signaling pathway during invasive candidiasis facilitates *C. glabrata* infection.

## RESULT

### The increased expression of MAPKs Fus3 and Kss1 during *C. glabrata* infection in the host

**Fig 1:**
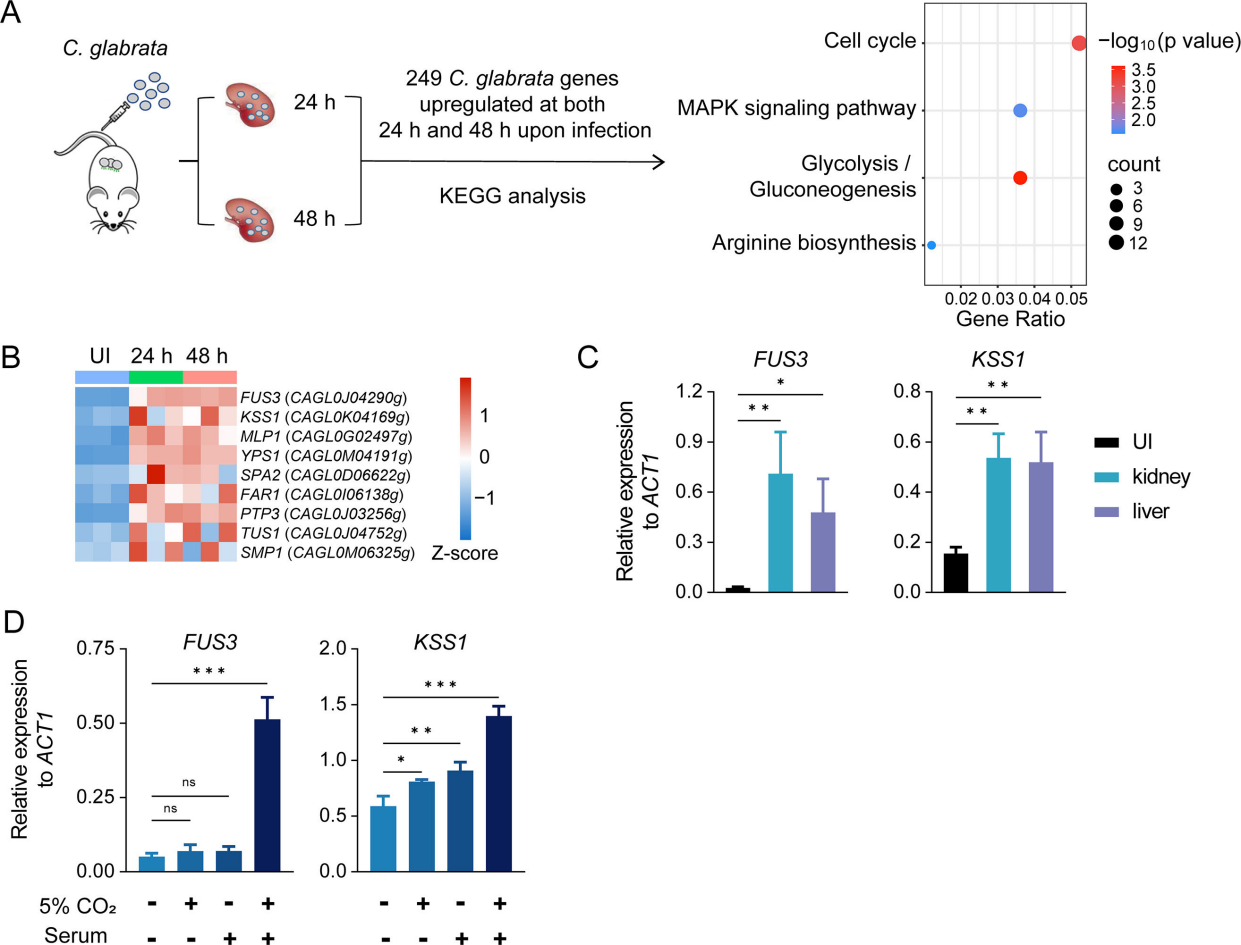
The expressions of MAPKs *FUS3* and *KSS1* are activated upon invasive infection of *C. glabrata*. (**A**) KEGG analysis of genes upregulated at both 24 h and 48 h during *C. glabrata* infection was performed using KOBAS (http://bioinfo.org/kobas/). (**B**) Heatmap showing the expression levels of genes for MAPK signaling pathway in (**A**), in z-score normalized to fragments per kilobase per million mapped reads (FPKM). UI, uninfected. (**C**) qRT-PCR analysis for the expression of *FUS3* and *KSS1* during invasive infection of *C. glabrata*. WT *C. glabrata* cells were grown in liquid YPD medium at 30°C for an *in vitro* control and then subjected to 19–21 g male BABL/c mice by tail vein injection. At 24 h post-infection, total RNA of the infected kidneys and livers was extracted for the *in vivo* analysis. (**D**) qRT-PCR analysis of *FUS3* and *KSS1* mRNA in WT *C. glabrata* cells incubated in DMEM medium with or without 10% serum in the absence or presence of 5% CO_2_ at 37°C. (C and D) The signals obtained from *ACT1* mRNA were used for normalization. Error bars represent standard deviations from the means of three experiments. Significance was measured with an unpaired *t*-test in GraphPad Prism. ns, no significance; *, *P*  <  0.05; **, *P*  <  0.01; ***, *P*  <  0.001.

To uncover the signaling pathway contributing to the virulence of *C. glabrata*, we performed *in vivo* RNA sequencing. Because the kidney is frequently the most heavily infected organ with hematogenously disseminated candidiasis, the RNA-seq analysis was applied to this organ at 24 h and 48 h post-infection (pi) (Fig. 1A, *n* = 3 mice). For each sample, a minimum of 65 million mRNA-Seq reads were mapped to the *C. glabrata* genome, which covered roughly 96% of the predicted genome. Cells grown under *in vitro* growth conditions (YPD 30°C) were used as a control to identify differentially expressed genes during *in vivo* infection. Two hundred and forty-nine genes upregulated in *C. glabrata* cells by 2-fold or more at both 24 h and 48 h pi (Table S1). These activated genes throughout the prompt response of *C. glabrata* upon infection (24 h and 48 h) were then subjected to KEGG analysis. As shown in Fig. 1A, they were significantly enriched in genes for glycolysis/gluconeogenesis (*P* = 2.49 × 10^−4^), cell cycle (*P* = 7.67 × 10^−4^), MAPK signaling pathway (*P* = 0.0199), and arginine biosynthesis (*P* = 0.0266). Among them, we focused on *CAGL0J04290g* and *CAGL0K04169g* (Fig. 1B). These two genes encode proteins sharing a high sequence homology with Fus3 and Kss1, two MAPKs in *S. cerevisiae* (Fig. 1A and B). We therefore designated *CAGL0J04290g* as *FUS3* and *CAGL0K04169g* as *KSS1* in *C. glabrata*.

The increased expression of *FUS3* and *KSS1* in murine kidneys during the invasive infection relative to *in vitro* growth condition (YPD medium) was confirmed by qRT-PCR (Fig. 1C). No significant difference was observed in the expression of *FUS3* and *KSS1* in RPMI medium compared with that in YPD medium (Fig. S2). In addition to kidneys, the enhanced expression of *FUS3* and *KSS1* was also observed in the infected livers, suggesting that host-associated signals might account for the *in vitro–in vivo* difference observed in the *FUS3* and *KSS1* transcripts. Indeed, *FUS3* and *KSS1* showed increased expression in response to host-associated signals, such as a combination of serum and high CO_2_ (Fig. 1D). Interestingly, serum or high CO_2_ alone was not sufficient to induce *FUS3* expression, indicating that *FUS3* expression is synergistically regulated by multiple signals within the host. Taken together, our transcription profiling data suggest that the MAPK signaling pathway is activated during *C. glabrata* infection.

### The MAPKs Fus3 and Kss1 are critical for the pathogenicity of *C. glabrata*

**Fig 2:**
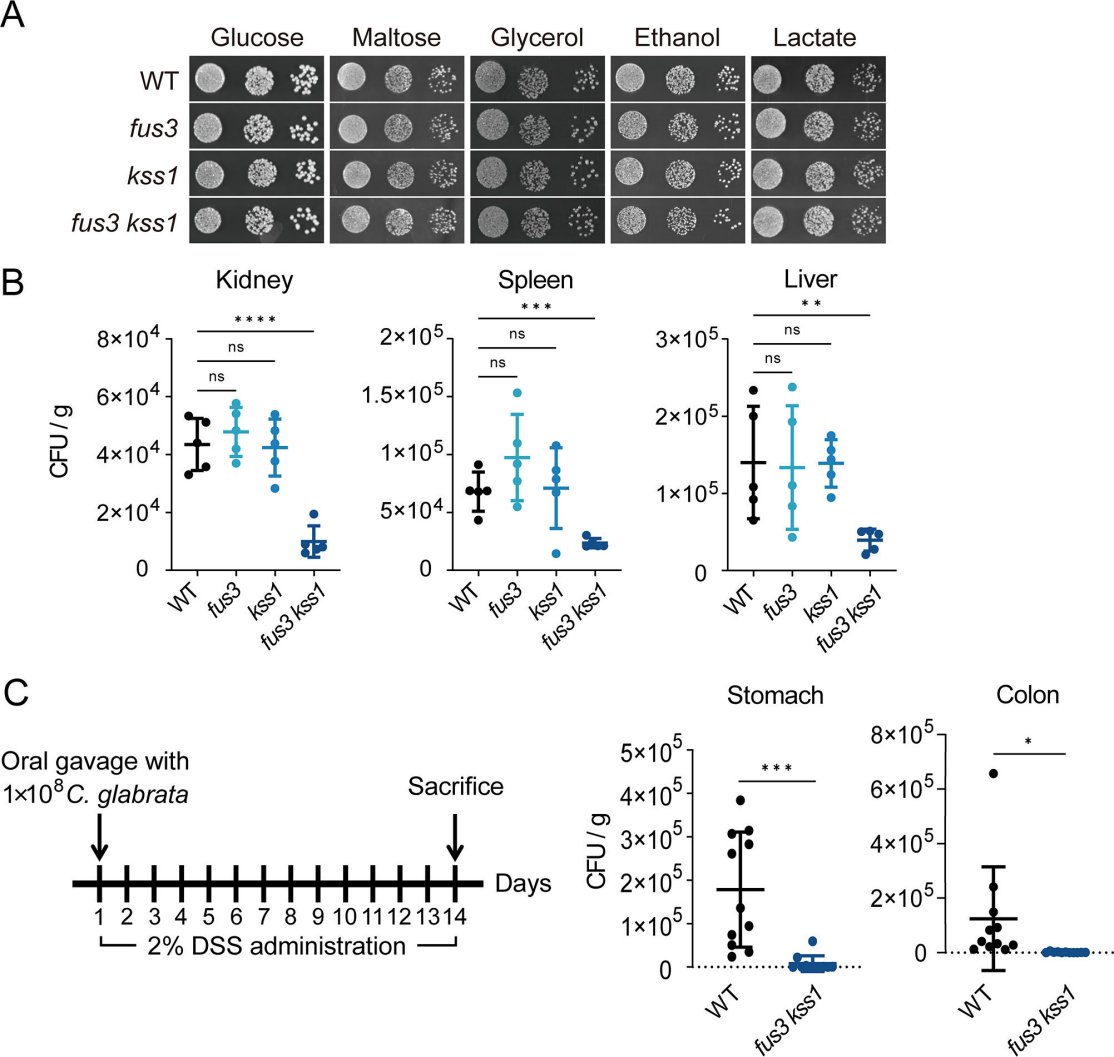
The *fus3 kss1* double mutant strain is defective in the persistence in infected organs of mice. (**A**) Cells of wild-type, single mutants *fus3* and *kss1*, and double mutant *fus3 kss1* were serially diluted 10-fold and spotted onto YNB solid medium containing 2% of the indicated carbon sources and incubated at 30°C. (**B**) Groups of immunosuppressed male BALB/c mice were infected with 5 × 10^7^ CFU of wild-type, *fus3* single mutant, *kss1* single mutant, or *fus3 kss1* double mutant by tail vein injection, followed by euthanasia of five animals per group after 4 days. BALB/c mice were rendered neutropenic by intraperitoneal administration of cyclophosphamide (150 mg/kg of body weight per day) 3 days before challenge with *C. glabrata* and on the day of infection. CFUs were determined by plating kidney, liver, or spleen homogenates onto agar plates (supplemented with streptomycin and ampicillin) and counting after incubation at 30°C. (**C**) The numbers of live *C. glabrata* recovered from the stomach and colon of mice during the induction of colitis by low doses of DSS. Eight- to 10-week-old female c57 mice were infected by oral gavage with 1 × 10^8^ cells of wild-type or *fus3 kss1* double mutant and treated with 2% DSS on the day of infection. The DSS administration occurred over a period of 2 weeks. Schematic overview of the protocol for the induction of colitis by DSS is shown on the left.*The values represent means ± SD for n* = 11 mice in each group. (B and C) Significance was measured with an unpaired *t*-test in GraphPad Prism. ns, no significance; *, *P*  <  0.05; **, *P*  <  0.01; ***, *P*  <  0.001; ****, *P*  <  0.0001.

To elucidate the impact of MAPK pathway on *C. glabrata* virulence, *fus3* and *kss1* single mutants were constructed. Since Fus3 and Kss1 are two paralogous MAPKs that have overlapping functions in pheromone response in *S. cerevisiae* (31), we also constructed *fus3 kss1* double mutant strain to further investigate their roles. It is found that single mutants *fus3* and *kss1,* and double mutant *fus3 kss1* exhibited no obvious growth defect in yeast extract-peptone glucose (YPD) liquid medium and YNB glucose plates at 30°C (Fig. S3; Fig. 2A). Considering the complex carbon sources in host niches, we next examined the growth of these mutants in fermentable carbon source maltose and nonfermentable carbon sources glycerol and ethanol, as well as the short-chain organic acid such as lactate. As shown in Fig. 2A, these mutants grew well on media containing indicated carbon sources.

We further determined the *C. glabrata* survival in the host during the invasive infection. Tissue fungal burdens were measured 4 days after infection in the immunosuppressed mice using tail-vein injections according to the previously established protocol (32). Mice infected with the *fus3 kss1* double mutant, but not *fus3* or *kss1* single mutant, had significantly lower fungal loads in the kidneys (~75%), livers (~70%), and spleens (~65%) than those infected with the WT strain (Fig. 2B). We next investigated the impact of Fus3 and Kss1 on the colonization of *C. glabrata* in the intestinal tract. Since all reported *C. glabrata* strains are eliminated within 2 days after challenge from mice without colitis in terms of fungal colonization, the DSS (Dextran Sulfate Sodium)-induced colitis model was used to experimentally mimic chronic inflammatory bowel diseases as reported previously with minor modification (18, 33). BALB/c mice were treated with 2% DSS on the day of infection with *C. glabrata* by oral gavage (Fig. 2C). Significantly higher numbers (>8-fold) of viable *C. glabrata* were detected in the stomach and colon of mice colonized with the WT strain than in mice colonized with the *fus3 kss1* double mutant strain after treatment with DSS for 14 d. Together, our data revealed a crucial role of Fus3 and Kss1 in *C. glabrata* survival in the host.

### The role of MAPKs Fus3 and Kss1 in *C. glabrata* survival in macrophages

**Fig 3:**
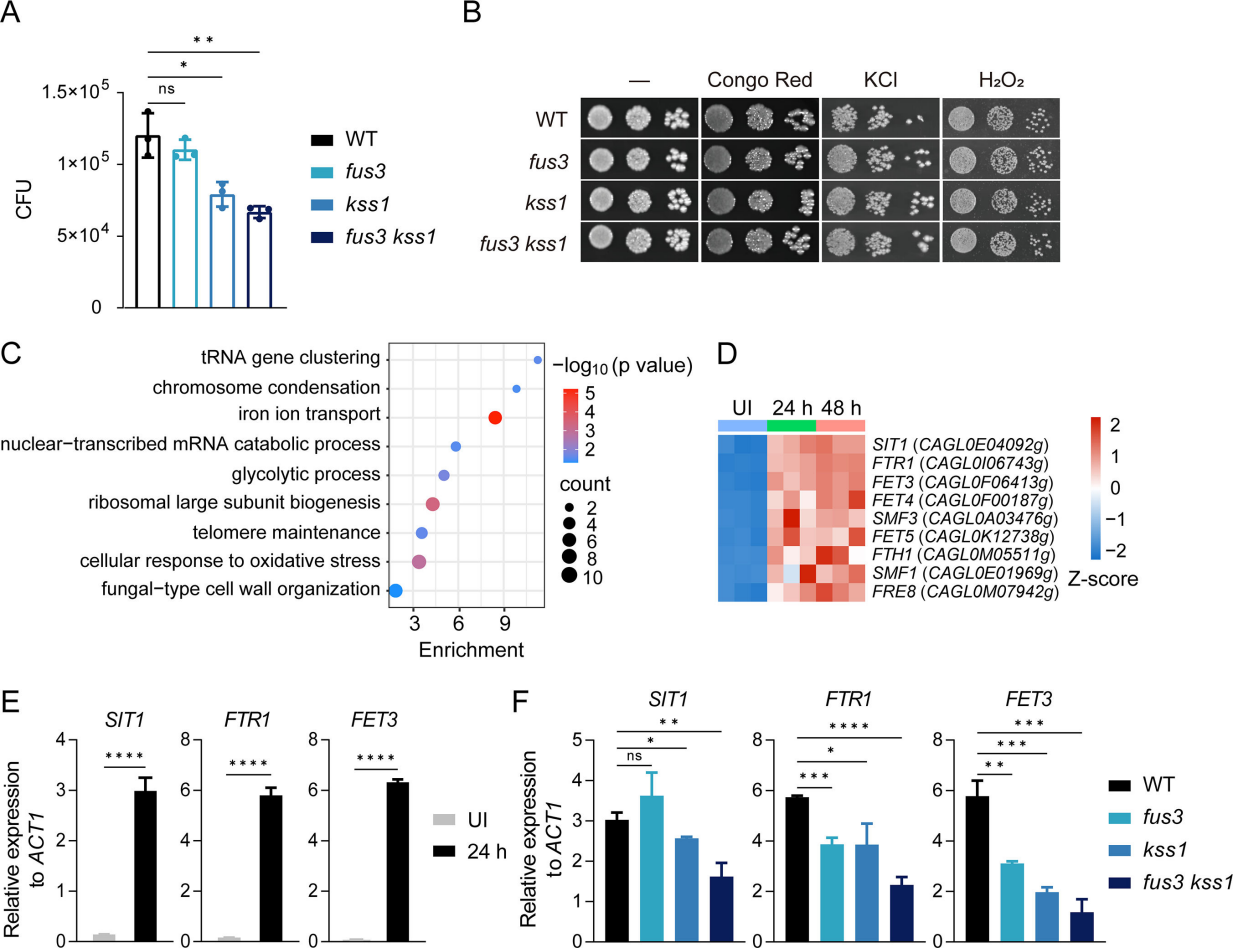
The impact of MAPKs Fus3 and Kss1 on the survival of *C. glabrata* in macrophages. (**A**) RAW264.7 cells were cultured with wild-type, single mutants *fus3* and *kss1*, and double mutant *fus3 kss1*. Non-phagocytosed *C. glabrata* cells were removed by washing with PBS after 3 h, and the CFUs of *C. glabrata* in RAW264.7 cells were determined after co-incubation for an additional 5 h. *n* = 3 biologically independent samples. (**B**) The MAPKs Fus3 and Kss1 are not implicated in stress responses in *C. glabrata*. Wild-type and indicated mutant strains were treated with stress agents, including 200  µg/mL Congo red, 1.5 M KCl, and 10 mM H_2_O_2_. Photographs were taken after growth at 30°C. (**C**) Gene Ontology (GO) biological process enrichment analysis of *C. glabrata* genes that are activated at both 24 h and 48 h upon invasive infection was performed using David (https://david.ncifcrf.gov/). (**D**) Heatmap showing the expression levels of genes for iron ion transport in (**C**), in z-score normalized to FPKM. UI, uninfected. (**E**) qRT-PCR analysis for the expression of *SIT1*, *FTR1,* and *FET3* in WT *C. glabrata* cells under *in vitro* and *in vivo* conditions was performed as described in Fig. 1C. At 24 h post-infection, total RNA of the infected kidneys was extracted for the *in vivo* analysis. (**F**) qRT-PCR analysis for the expression of *SIT1*, *FTR1,* and *FET3* in wild-type, single mutants *fus3* and *kss1*, and double mutant *fus3 kss1*. The *C. glabrata* cells were subjected to male BALB/c mice by tail vein injection. Total RNA was extracted from the kidneys after 24 h of infection. (E and F) The mRNA levels of the indicated genes were normalized with *ACT1*. Mean data ± SD from three independent experiments were plotted. Significance was measured with an unpaired *t*-test in GraphPad Prism. ns, no significance; *, *P*  <  0.05; **, *P*  <  0.01; ***, *P*  <  0.001; ****, *P*  <  0.0001.

To understand the mechanism by which MAPKs regulate *C. glabrata* virulence, we first investigated the fungistatic activity of macrophages toward wild-type, single mutants *fus3* and *kss1,* and double mutant *fus3 kss1*. Interestingly, a significant reduction (~35%) in the fungal burden was observed in *kss1* single mutant compared with the wild-type after exposure to macrophages for 8 h (Fig. 3A), although this mutant exhibited no phenotype for the survival in the infected organs during invasive infection (Fig. 2B), suggesting that multiple virulence factors may work together to determine the *C. glabrata* virulence in the host. Furthermore, the *fus3 kss1* double mutant displayed a more severe deficiency in resistance to intracellular killing by phagocytic cells than the *kss1* single mutant (Fig. 3A). However, all these mutants grew normally upon stress challenges under the *in vitro* condition, including osmotic stress (KCl), oxidative stress (H_2_O_2_), and cell wall stress (Congo red) (Fig. 3B). One unresolved question is how the MAPK signaling pathway influences the survival of *C. glabrata* within the macrophages.

*C. glabrata* has developed effective strategies to sequester iron from host cells, which is critical for its pathogenicity (34). The siderophore-iron transporter Sit1 and the high-affinity iron permease Ftr1 have been reported to be required for Fe acquisition and survival of *C. glabrata* during macrophage infection (35–37). Indeed, we found that genes activated throughout the prompt response of *C. glabrata* upon infection (24 h and 48 h) were enriched for gene ontology (GO) terms associated with iron ion transport (*P* = 6.05 × 10^−6^), including *SIT1* and *FTR1* (Fig. 3C and D). Also, the activation of *FET3* encoding the oxidase that oxidizes ferrous (Fe^2+^) to ferric iron (Fe^3+^) for subsequent cellular uptake by transmembrane permease Ftr1 was identified during the invasive infection of *C. glabrata* (Fig. 3D). The upregulation of *SIT1*, *FTR1,* and *FET3* at 24 h post-invasive infection was then confirmed by qRT-PCR (Fig. 3E). Interestingly, the upregulation of *SIT1* gene during invasive infection was dependent on Kss1 but less dependent on Fus3 (Fig. 3F). In contrast, both Fus3 and Kss1 were critical for the upregulation of *FTR1* and *FET3* (Fig. 3F). Therefore, MAPKs Fus3 and Kss1 regulate the expression of genes for iron ion transport in a coordinated and complementary manner.

### MAPKs Fus3 and Kss1 regulate biofilm formation and adhesion to human epithelial cells in *C. glabrata*

**Fig 4:**
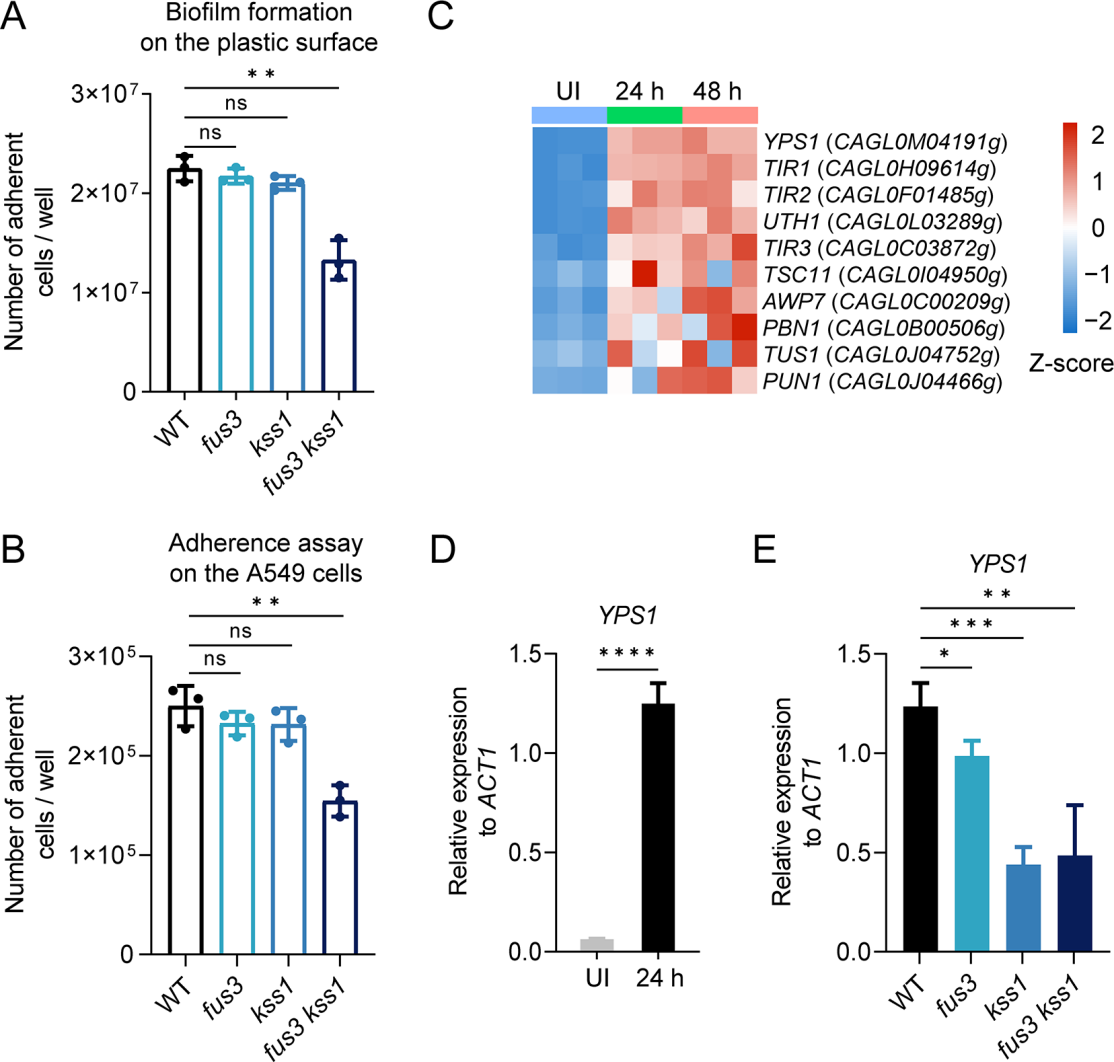
Deletion of MAPKs Fus3 and Kss1 caused a reduction in capacity for adhesion of *C. glabrata*. (**A**) Cells of wild-type, single mutants *fus3* and *kss1*, and double mutant *fus3 kss1* were inoculated onto 48-well plates with 500 µL SDB medium at 37°C for 48 h. The formation of biofilm was analyzed by the number of adherent cells. Values are the means ± SD from three independent experiments. (**B**) Adhesion assay on epithelial cell monolayers. *C. glabrata* cells of wild-type and indicated mutant strains were inoculated with A549 cells in DMEM medium supplemented with 10% serum. After incubation for 4 h, non-adherent *Candida* cells were removed by washing with PBS. The numbers of adherent *Candida* cells were represented as means ± SD from three independent experiments. (**C**) Heatmap showing the expression levels of genes for fungal-type cell wall organization in Fig. 3C, in z-score normalized to FPKM. UI, uninfected. (**D**) qRT-PCR analysis for the *YPS1* expression in WT *C. glabrata* cells under *in vitro* and *in vivo* conditions was performed as described in Fig. 1C. At 24 h post-infection, total RNA of the infected kidneys was extracted for the *in vivo* analysis. (**E**) qRT-PCR analysis for the expression of *YPS1* in wild-type, single mutants *fus3* and *kss1*, and double mutant *fus3 kss1* during invasive infection. Total RNA was extracted from the kidneys after 24 h of infection. (D and E) The mRNA levels of *YPS1* were normalized with *ACT1*. Mean data ± SD from three independent experiments were plotted. Significance was measured with an unpaired *t*-test in GraphPad Prism. ns, no significance; *, *P*  <  0.05; **, *P*  <  0.01; ***, *P*  <  0.001; ****, *P*  <  0.0001.

The biofilm represents a major form of resistance to host defense machinery during fungal infection, we therefore placed *C. glabrata* cells in a 48-well plate to allow the biofilm formation. Similar biofilm formation was observed among the wild-type, *fus3* mutant, and *kss1* mutant on the plastic surface of the wells (Fig. 4A). However, the number of adherent cells in the *fus3 kss1* double mutant was approximately half of that observed in wild-type strain, suggesting that deletion of MAPKs Fus3 and Kss1 resulted in a decreased adhesion to the plastic surface in *C. glabrata* (Fig. 4A). Furthermore, the adherence assay on the human epithelial cells A549 revealed that *fus3 kss1* double mutant exhibited significantly reduced (~35%) attachment compared with the wild-type (Fig. 4B). Therefore, our results provide evidence for the overlapping role of Fus3 and Kss1 on the regulation of adherence to materials or cellular surfaces in *C. glabrata*. The Epithelial Adhesin (EPA) family has been found to enable *C. glabrata* to attach to epithelial cells (12, 15). However, the *in vivo* infection did not trigger a significant change in the expression of these EPA adhesins based on our transcription profiling data. Instead, 10 genes involved in the fungal-type cell wall organization (*P* = 0.0493) showed a dramatically increased expression at both 24 h and 48 h post-infection (Fig. 3C and 4C). Among them, the YPS-family aspartyl protease Yps1 plays a role in remodeling *C. glabrata* cell wall by removal of Epa1, which is largely responsible for the *in vitro* adherence to epithelial cells (16). In fact, a specific requirement for Yps1 in biofilm formation has been identified in *C. glabrata* (38). As shown in Fig. 4D, the induced expression of *YPS1* during invasive infection of *C. glabrata* was confirmed by qRT-PCR. Remarkably, we found that Fus3 and Kss1 are both involved in the transcriptional activation of *YPS1* during *in vivo* infection (Fig. 4E). Thus, our data suggested that Fus3 and Kss1 may regulate the organization of cell wall proteins, such as the adhesin Epa1, to promote adherence of *C. glabrata.*

We next wanted to determine whether constitutive expression of *FUS3* or *KSS1* can lead to an increase in the adherence capacity of *C. glabrata*. As shown in Fig. S4A, the biofilm formation was not activated upon *FUS3* or *KSS1* overexpression. Also, overexpression of *FUS3* or *KSS1* under the *TEF1* promoter failed to promote the adhesion to human epithelial cells (Fig. S4B) and was unable to increase the fungal burden in murine kidneys during invasive infection (Fig. S4C). Our data indicated that the transcriptional upregulation of *FUS3* and *KSS1* is insufficient to promote pathogenicity. Rather, we suggest that the activation of these two MAPKs relies on the phosphorylation event, although this hypothesis needs further study.

### Downstream transcription factors of MAPK signaling pathway are implicated in *C. glabrata* pathogenicity

**Fig 5:**
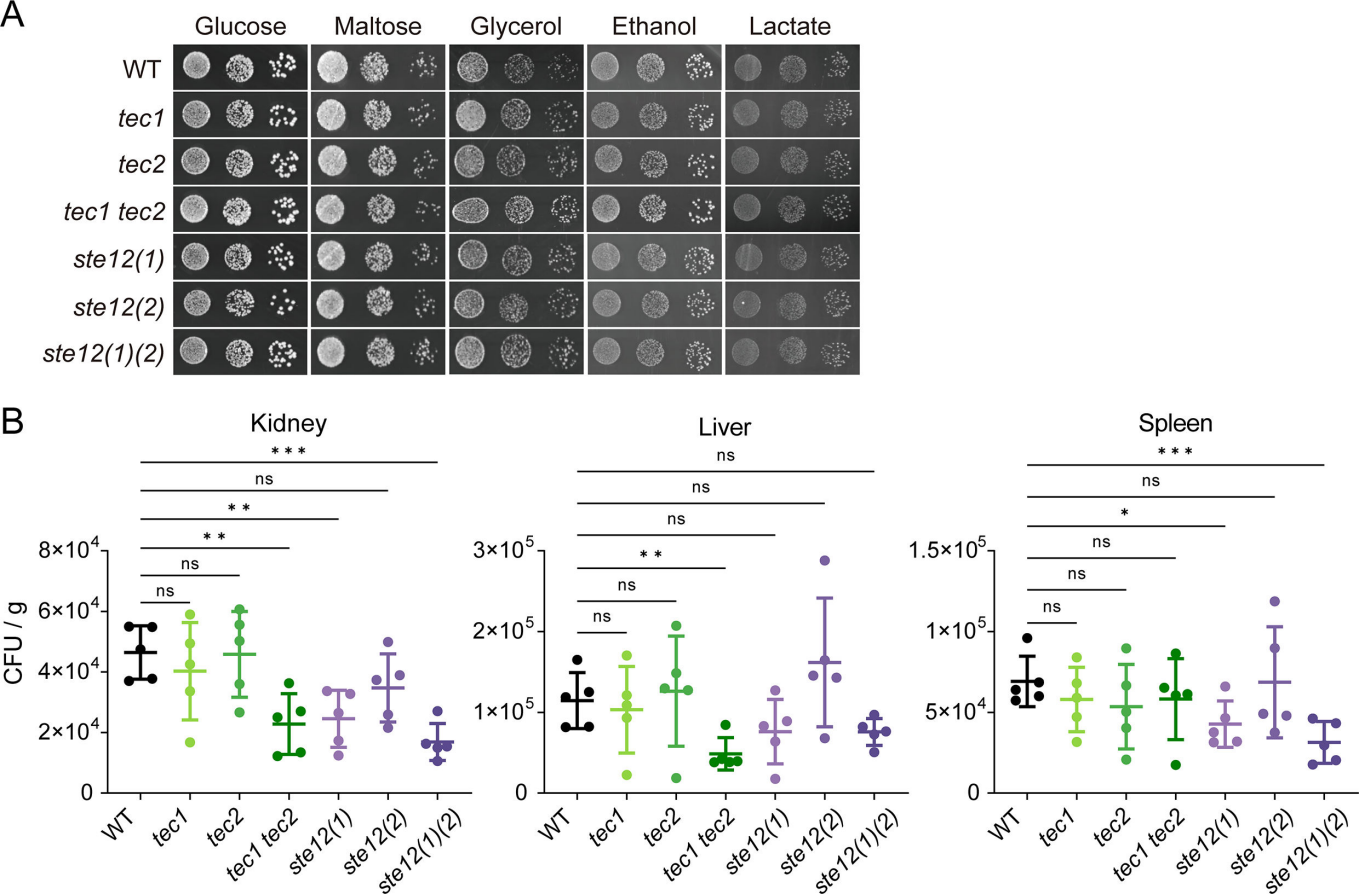
Ste12 and Tec1 homologs impact the virulence of *C. glabrata* during disseminated infection in mice. (**A**) Dilutions of wild type and indicated mutant strains were spotted onto YNB solid medium containing 2% glucose, maltose, glycerol, ethanol, or lactate and incubated at 30°C. (**B**) The fungal loads of wild type, single mutants *tec1*, *tec2*, *ste12 (1),* and *ste12 (2)*, and double mutants *tec1 tec2* and *ste12(1)(2)* in immunosuppressed mice were determined as in Fig. 2B. *n* = 5 mice. Significance was measured with an unpaired *t*-test in GraphPad Prism. ns, no significance; *, *P*  <  0.05; **, *P*  <  0.01; ***, *P*  <  0.001.

The downstream targets of MAPKs include the transcription factors Ste12 and Tec1, both of which are required for the expression of filamentation genes in *S. cerevisiae* (26). Although only one copy of *STE12* or *TEC1* is presented in *S. cerevisiae* genome, they are both duplicated in *C. glabrata* (39). We therefore deleted each of them to obtain four single mutant strains, including *tec1* (*CAGL0M01716g*), *tec2* (*CAGL0F04081g*), *ste12(*1*)* (*CAGL0M01254g*), and *ste12(*2*)* (*CAGL0H02145g*). Considering the evolutionary relationship of the homologous genes, we also constructed the *tec1 tec2* and the *ste12(1)(2)* double mutant strains. These single mutant strains *tec1*, *tec2*, *ste12 (1),* and *ste12 (2),* and double mutant strains *tec1 tec2* and *ste12 (1) ste12 (2)* grew well on rich medium (YPD medium), similar to the WT strain (Fig. S5). Also, these mutant cells displayed no obvious growth defect in fermentable carbon source maltose and nonfermentable carbon sources glycerol, ethanol, and lactate (Fig. 5A).

The contribution of Ste12 and Tec1 to the *C. glabrata* virulence was then determined using the model of hematogenously disseminated candidiasis. As shown in Fig. 5B, the single mutant strain *ste12 (1)* and double mutant strains *tec1 tec2* and *ste12 (1) ste12 (2)* showed severely attenuated fungal burden in the kidney during the invasive infection. Also, *tec1 tec2* double mutant showed a notable decrease in the persistence within the liver. Although we could not detect a statistically significant difference in fungal burden of infected livers between the strains deleting *STE12* homologs and the WT strain, the *ste12 (1)* single mutant and *ste12 (1) ste12 (2)* double mutant exhibited an observable reduction in survival ability in the liver compared with the WT strain (Fig. 5B). In fact, Ste12 (1) has been identified to be required to maintain wild-type levels of virulence in a reported murine model of *C. glabrata* systemic disease (22). Here, we found that the *ste12(1)(2)* double mutant displayed a more severe survival deficiency in infected kidneys than the *ste12 (1)* single mutant, suggesting that Ste12 homologs, Ste12 (1) and Ste12 (2), may have distinct functions during the invasive infection of *C. glabrata.* Moreover, mice infected with the *tec1 tec2* double mutant strain had significantly lower fungal burdens compared with those infected with the WT strain or single mutants, *tec1* and *tec2* (Fig. 5B), indicating the overlapping functions of Tec1 and Tec2 in modulating *C. glabrata* virulence. Interestingly, only *ste12 (1)* single mutant and *ste12 (1) ste12 (2)* double mutant exhibited significantly lower fungal loads in the spleen (Fig. 5B), suggesting that *C. glabrata* MAPKs Fus3 and Kss1 may employ distinct downstream transcription factors to adapt and survive in different host niches during infection.

### The regulation of Ste12 and Tec1 homologs in the adhesion and iron transport of *C. glabrata*

**Fig 6:**
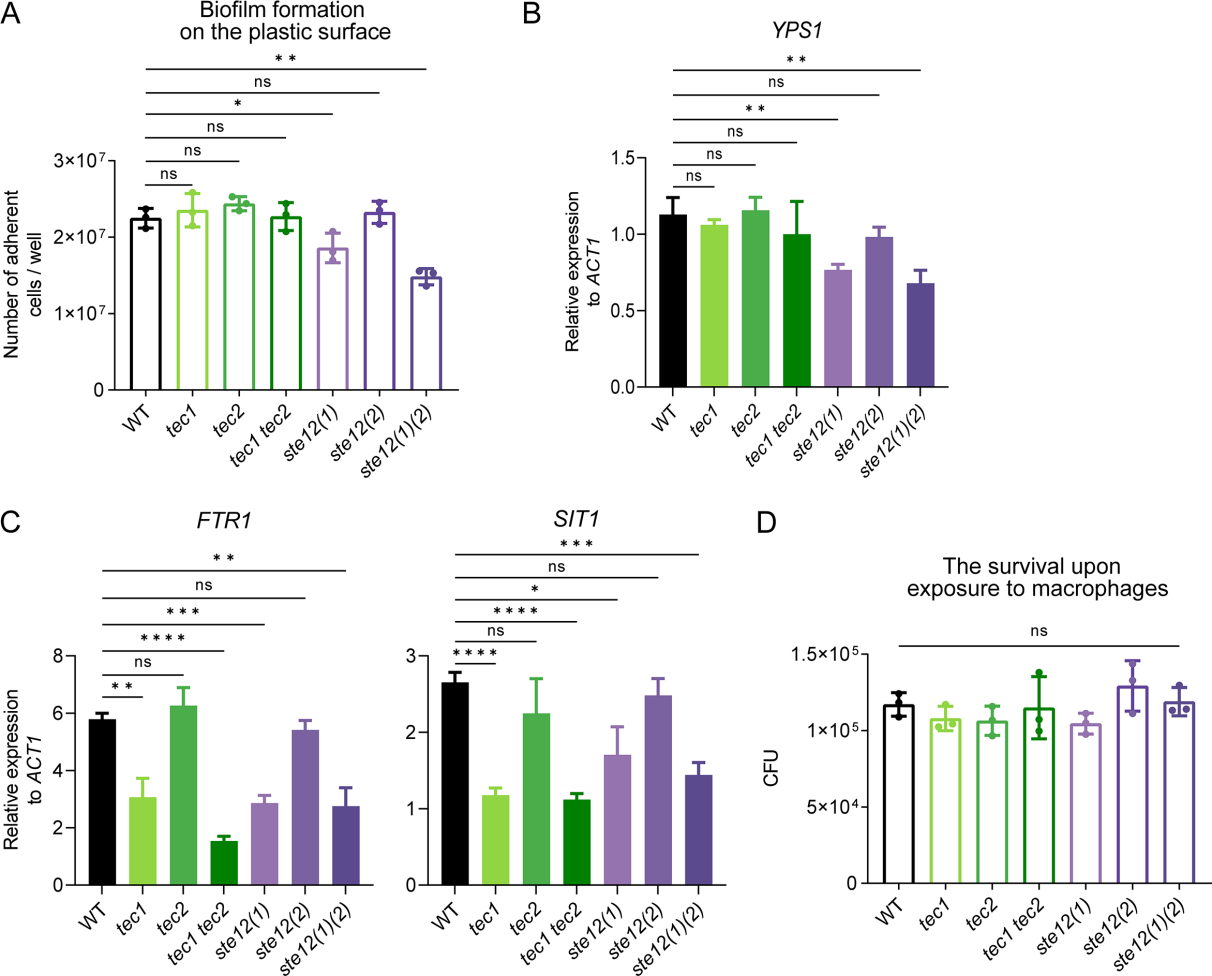
Functional characterization of Ste12 and Tec1 homologs in *C. glabrata*. (**A**) The formation of biofilm for wild-type, single mutants *tec1*, *tec2*, *ste12 (1)*, and *ste12 (2)*, and double mutants *tec1 tec2* and *ste12(1)(2)* was analyzed as described in Fig. 4A. Values are the means ± SD from three independent experiments. qRT-PCR analysis for the expression of *YPS1* (**B**), *FTR1* (**C**), and *SIT1* (**C**) in wild-type and indicated mutant strains. *C. glabrata* cells were introduced into male BALB/c mice through tail vein injection. After 24 h of infection, total RNA was extracted from the kidneys. The mRNA levels of the indicated genes were normalized with *ACT1*. (**D**) The survival of mutant cells with the deletion of *STE12* or *TEC1* homologs was similar to that of wild-type after exposure to macrophages. The quantification of *C. glabrata* cells that survived within macrophages was carried out as detailed in Fig. 3A. *n* = 3 biologically independent samples. (**A-D**) Significance was measured with an unpaired *t*-test in GraphPad Prism. ns, no significance; *, *P*  <  0.05; **, *P*  <  0.01; ***, *P*  <  0.001; ****, *P*  <  0.0001.

The biofilm formation assay was performed in mutant strains deleting *STE12* or *TEC1* homologs and revealed that *ste12 (1)* single mutant and *ste12 (1) ste12 (2)* double mutant exhibited reduced attachment compared with the wild-type (Fig. 6A), which is consistent with a previous study (23). Correspondingly, a reduction in the expression of *YPS1* was observed in these mutants compared with WT strain during invasive infection (Fig. 6B). Our result suggested that the activation of *YPS1* by MAPKs Fus3 and Kss1 might be mediated through Ste12 homologs. However, both Ste12 and Tec1 homologs were implicated in the regulation of iron acquisition as a lower *SIT1* and *FTR1* expression was observed in the *ste12 (1) ste12 (2)* or *tec1 tec2* double mutant than that in WT strain during *in vivo* infection (Fig. 6C). We next investigated the fungistatic activity of macrophages toward mutant strains lacking *STE12* or *TEC1* homologs. Unexpectedly, no significant difference in fungal burden was observed in these mutant strains compared with the wild-type after exposure to macrophages for 8 h (Fig. 6D), suggesting the involvement of other downstream effectors of MAPKs Fus3 and Kss1 in the *C. glabrata* resistance to intracellular killing by phagocytic cells.

### Deletion of MAPKs Fus3 and Kss1 enhanced caspofungin efficacy against *C. glabrata.*

**Fig 7:**
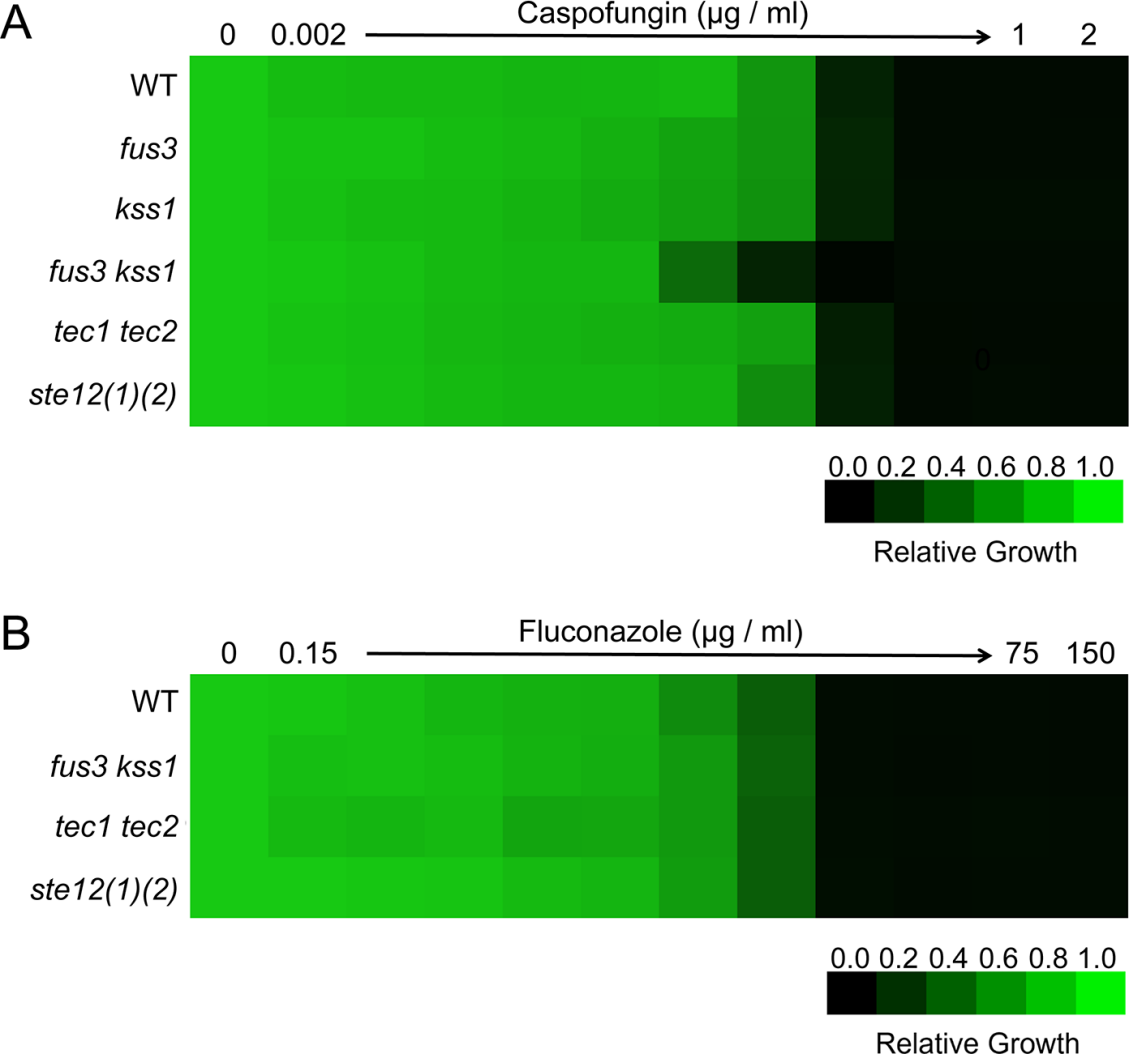
Caspofungin (**A**) and fluconazole (**B**) susceptibility assays were conducted in the YPD medium for wild-type and indicated mutant strains. Growth was measured by absorbance at 600  nm after 24  h at 30°C. Optical densities were normalized relative to those of antifungal drug-free controls. Data are quantitatively displayed in heat map format (see color bar).

The echinocandins, including caspofungin, represent the first-line drugs for the treatment of systemic candidiasis, exerting their antifungal effect by targeting β−1,3-glucan synthase Fks1. It has been reported that the *S. cerevisiae* Fus3 homolog in *C. albicans* (Cek1) is activated in response to caspofungin treatment (40). We therefore determined the impact of MAPKs and downstream transcription factors in the susceptibility to caspofungin in YPD liquid medium containing caspofungin with different concentrations. As shown in Fig. 7A, only *fus3 kss1* double mutant displayed a slightly increased susceptibility to caspofungin compared with that of WT *C. glabrata*. Fus3 and Kss1 may act in a synergistic manner for caspofungin tolerance as the *fus3* and *kss1* single mutants grew similarly to WT cells in a caspofungin-containing medium. The *tec1 tec2* and *ste12(1)(2)* double mutant strains displayed no obvious defects in caspofungin response (Fig. 7A), implying that other downstream factors of MAPKs are responsible for caspofungin tolerance. In contrast, deletion of *FUS3* and *KSS1* had little effect on the sensitivity to fluconazole (Fig. 7B), suggesting that Fus3 and Kss1 MAPKs are specifically required for echinocandin tolerance. Consistently, the growth rate of *tec1 tec2* and *ste12 (1) ste12 (2)* double mutant strains was comparable with that of WT strain in a fluconazole-containing medium (Fig. 7B).

## DISCUSSION

The mitogen-activated protein (MAP) kinase pathways are important in mediating responses to diverse extracellular signals in fungi and other eukaryotic organisms. A classical MAP kinase cascade comprises a MAP kinase (MAPK), a MAPK kinase (MEK), and a MEK kinase (MEKK) (41). The MAPKs target downstream proteins, influencing transcriptional events and cellular behaviors (42). There are five MAPK genes (*FUS3*, *KSS1*, *SLT2*, *HOG1*, and *SMK1*) in the model organism *S. cerevisiae*. Two of them (*FUS3* and *KSS1*) are closely related and have overlapping functions in pheromone response. In addition, Kss1 contributes to filamentation and invasive growth into agar (31). Slt2 and Hog1 MAPKs predominantly not only govern cell wall integrity and osmoregulation, respectively, but also participate in regulating responses to other stresses (43, 44). Recently, it has been found that surplus-extracellular iron activates the MAPK Hog1, resulting in the transcriptional activation of the adhesin Epa1 in *C. glabrata* (45). Smk1 is a meiosis-specific MAPK regulating ascospore wall assembly (46).

Here, we found that the expression of *FUS3* and *KSS1* underwent a significant increase throughout the prompt response during *C. glabrata* infection. Interestingly, the induction patterns of *FUS3* and *KSS1* differ in response to host environmental signals (Fig. 1D). Both serum and high CO_2_ contributed to *FUS3* induction, but neither alone was sufficient. However, the activation of *KSS1* expression could be observed in either serum or high CO_2_. The mechanisms underlying environmental cues that activate the MAPK signaling pathway need to be further investigated in *C. glabrata*. In addition, an increase in the *SLT2* expression was identified in *C. glabrata* cells at 24 h post-infection in our transcription profiling data, although no significant difference was detected at 48 h post-infection (Zhang and Lu, submitted for publication). Indeed, the increased *SLT2* expression has been found to facilitate the survival of *C. glabrata* cells in host tissues, perhaps due to increased tolerance to stressful conditions that affect cell wall integrity (47). It is worth noting that the activation of MAPK signaling pathway might be not only mediated by inducing the expression of MAPKs during *C. glabrata* disseminated infection since the constitutively overexpressed *FUS3* and *KSS1* have no effect on the fungal survival in the infected kidneys (Fig. S4C). In fact, phosphorylation is a common event for signal transduction in MAPK signaling pathway.

MAPKs Slt2 and Hog1 have been extensively studied in *C. glabrata*. Slt2 MAPK pathway is found to be implicated in the heat-induced expression of *YPS1*, which is essential for cell wall integrity and virulence in *C. glabrata* (16, 48). Although Hog1 is not required for virulence in a murine model of systemic infection, it plays an important role in the confrontation of *C. glabrata* with the common vaginal flora (49). Because most fungal pathogens have more complicated lifestyles and differentiation processes, it is reasonable to hypothesize that these MAP kinases may play more diverse roles in pathogenic fungi compared with their counterparts in *S. cerevisiae*. Several lines of evidence provided in our study support the role of MAPKs Fus3 and Kss1 as important regulators for the *C. glabrata* pathogenicity. First, the deletion of both *FUS3* and *KSS1* resulted in a profound defect in the fungal survival of *C. glabrata* during the invasive infection, suggesting the redundant role of Fus3 and Kss1 on *C. glabrata* infection. However, Fus3 and Kss1 also have different functions regarding *C. glabrata* physiology, as we noticed that Kss1 was required for the survival of *C. glabrata* within the macrophages, whereas Fus3 played a minor role in it (Fig. 3A). How Fus3 and Kss1 differentially regulate this process needs further study. Such overlapping and divergent roles of Fus3 and Kss1 were also found in *S. cerevisiae* in the regulation of pheromone response. Second, Fus3 and Kss1 contributed to the persistence of *C. glabrata* within macrophages, at least partially through the activation of genes involved in iron transport. Third, *fus3 kss1* double mutant exhibited reduced adherence to the abiotic surfaces and human epithelial cells. Importantly, the colonization was diminished in the *fus3 kss1* double mutant compared with the WT strain in a murine model of DSS-induced colitis (Fig. 2C). Fourth, Fus3 and Kss1 activate the expression of the aspartyl protease Yps1, which has been found to play an important role in remodeling *C. glabrata* cell wall (16). Whether and how Fus3 and Kss1 impact cell wall remodeling during *C. glabrata* invasive infection deserves further investigation. Interestingly, the cAMP/PKA pathway, rather than the MAPK pathway, plays a major role in the pathogenesis of *C. albicans* (50). For another important human pathogenic fungus *C. neoformans*, the Fus3 homolog does not contribute to the virulence (28). Nevertheless, we clearly demonstrate that the Fus3 and Kss1 MAP kinases, which play a minor role in the virulence of some pathogenic fungi, significantly impact the pathogenicity of *C. glabrata*. Our study provides an example of how a conserved signaling pathway rewires to regulate fungal pathogenesis.

The signal transduction pathways mediated by MAPKs Fus3 and Kss1 for mating and invasive growth in *S. cerevisiae* converge on the transcription factor Ste12. For invasive growth, both Ste12 and its cofactor Tec1 are indispensable (51–53). Interestingly, only one copy of *STE12* and *TEC1* are presented in *S. cerevisiae* genome, but they are doubled in *C. glabrata*, indicating that the transcriptional regulatory network in *C. glabrata* is more complex than in the model yeast *S. cerevisiae* (39). Indeed, the distinct contributions to virulence from Ste12 (1) and Ste12 (2) were observed in *C. glabrata*. Ste12 (1) seems to play a more significant role compared with Ste12 (2) in *C. glabrata* virulence since deletion of *STE12 (1)* alone is sufficient to cause a decrease in the fungal survival during invasive infection (Fig. 5B). Although there was no significant difference in fungal loads between wild-type and *ste12 (2)* single mutant strain during invasive infection, deleting *STE12 (2)* in the *ste12 (1)* mutant strain exacerbated the defect in virulence. This suggested that Ste12 (1) and Ste12 (2) may have partially overlapping functions in modulating the virulence of *C. glabrata*. In contrast, the attenuated survival of *C. glabrata* in infected organs was only detected when *TEC1* and *TEC2* were both deleted, indicating a synergistic effect between these two Tec1 homologs on *C. glabrata* virulence. Interestingly, no defect was observed in the *ste12 (1) ste12 (2)* or *tec1 tec2* double mutant in response to intracellular killing by phagocytic cells (Fig. 6D), in contrast to the lower burden of the *kss1 fus3* mutant in macrophages (Fig. 3A). In addition, unlike the *kss1 fus3* mutant, which was more susceptible to caspofungin, the susceptibility of *ste12 (1) ste12 (2)* and *tec1 tec2* mutant to caspofungin was comparable with WT (Fig. 7B). These results suggest that other factors are involved in these processes and this requires further research.

In addition to regulating virulence attributes in *C. glabrata*, Fus3 and Kss1 impact the tolerance to caspofungin, an anti-fungal drug of echinocandin class. In fact, deletion of the Fus3 ortholog MpkB has been reported to increase the susceptibility to caspofungin in *Aspergillus fumigatus* (54). Since echinocandins exert their antifungal effect by compromising the integrity of the fungal cell wall, these results suggested that homologs for MAPKs Fus3 and Kss1 may play a role in cell wall biosynthesis. The function of other MAPKs in drug tolerance, such as Slt2 and Hog1, has been investigated in *C. glabrata* in previous studies. Slt2 was found to play an important role in response to echinocandins, including caspofungin and micafungin (47, 55, 56). Hog1 alters the susceptibility to fluconazole, another anti-fungal drug commonly used to treat *Candida* infections (57). It would be intriguing to investigate how these diverse MAPK pathways orchestrate responses to antifungal drugs.

## MATERIALS AND METHODS

### Media and growth conditions

*C. glabrata* strains were routinely grown at 30°C in YPD medium (2% Bacto peptone, 2% glucose, 1% yeast extract). Transformants were selected on YPD plates supplemented with 80 µg/mL nourseothricin or synthetic medium (0.17% Difco yeast nitrogen base w/o ammonium sulfate, 0.5% ammonium sulfate, and auxotrophic supplements) with 2% glucose. The ability of *C. glabrata* cells to grow was tested by spotting dilutions of cells onto YNB (0.17% Difco yeast nitrogen base w/o ammonium sulfate, 0.5% ammonium sulfate) solid media with 2% of different sugars followed by incubation at 30°C.

To determine the stress response of *C. glabrata*, freshly grown cells were serially diluted 10-fold, spotted onto YPD plates with or without 200 µg/mL Congo red, 1.5 M KCl, or 10 mM H_2_O_2_, and incubated at 30°C.

### Plasmid and strain construction

CBS138 genomic DNA was used as the template for all PCR amplifications of *C. glabrata* genes. *C. glabrata* strains used in this study are listed in Table 1. The primers used for PCR amplifications are listed in Table S2.

**TABLE 1:** *C. glabrata* strains used in this study

Strains	Genotype	Source
CBS138	Wild-type	ATCC collection
YLC113	*fus3Δ*	This study
YLC114	*kss1Δ*	This study
YLC115	*fus3Δ kss1Δ*	This study
YLC116	*tec1Δ*	This study
YLC117	*tec2Δ*	This study
YLC118	*tec1Δ tec2Δ*	This study
YLC119	*ste12 (1)Δ*	This study
YLC120	s*te12 (2)Δ*	This study
YLC121	*ste12 (1)Δ ste12 (2)Δ*	This study
YLC111	*ura3Δ::SAT1*	This study

Deletion of *FUS3* (*CAGL0J04290g*), *KSS1* (*CAGL0K04169g*), *TEC1* (*CAGL0M01716g*), *TEC2* (*CAGL0F04081g*), *STE12(*1*)* (*CAGL0M01254g*), and *STE12(*2*)* (*CAGL0H02145g*) was performed using CRISPR-Cas9 strategy as follows. The single-guide RNA (sgRNA) was annealed to insert into the pV1382 vector (58). The resulting plasmid was transformed into *C. glabrata* cells with the repair template. The mutants were verified by sequencing.

The coding sequences for *FUS3* (Primers 37 and 38) and *KSS1* (Primers 39 and 40) were amplified from *C. glabrata* genomic DNA. The PCR product was then inserted into the NotI-SacII site of pY26TEF-GPD, generating the overexpressing plasmid to express *FUS3* or *KSS1* under the control of *TEF1p* in *C. glabrata*.

### *In vivo* RNA sequencing and analysis

For RNA-seq assay during *C. glabrata* infection, 6- to 8-week-old male BALB/c mice were inoculated with 4.5 × 10^8^ wild-type cells (CBS138) in a 200 µL volume of sterile PBS via tail vein injection. At 24 and 48 h post-infection, animals were euthanized. The infected kidneys from two mice were randomly picked from a certain group and combined as a sample for RNA extraction, with uninfected *C. glabrata* cells (UI) incubated in the YPD medium at 30°C as the control. DNA-depleted RNA samples were then depleted of ribosomal RNA using the Ribo-Zero rRNA Removal Kit (Epicentre) according to the manufacturer’s protocol. Sequencing was performed using the Illumina nova6000 platform. Clean reads were selected from raw reads by removing reads with adapter and low quality. Q30 and GC content of clean data were calculated. The sequencing reads were then aligned to *C. glabrata* reference genome (http://www.candidagenome.org/) using HISAT2 with default parameters, and the aligned reads were assembled and quantified. Genes with zero counts were excluded. Differentially expressed genes were defined by fold change ≥2 and a false discovery rate (FDR) < 0.05 was found by DESeq2. KEGG analysis was performed using KOBAS (http://bioinfo.org/kobas/). Gene Ontology (GO) biological process enrichment analysis was performed using David (https://david.ncifcrf.gov/).

**Biofilm assay**

Biofilm growth assays *in vitro* were conducted following a modified version of a previously described protocol (15). In brief, *C. glabrata* strains were cultured overnight in liquid YPD medium at 30°C. The cultures were washed twice using phosphate-buffered saline (PBS) and subsequently diluted in 500 µL of SDB medium (1% Bacto peptone, 4% glucose) to an optical density at OD_600_ of 0.1. This mixture was placed in the 48-well polystyrene plate and incubated at 37°C with a shaking speed of 70 rpm to allow the adhesion. After 48 h of incubation, the non-adherent cells were removed by washing twice with PBS, and the adherent cells were treated with proteinase K for 1 h. The quantification of adherent cells was performed through direct microscopic counting.

### Infection of macrophages

RAW264.7 cells were challenged with *C. glabrata* at a MOI of 1:1 (macrophage:*Candida*). Nonphagocytosed *Candida* cells were removed by washing with PBS after co-incubation for 3 h. To determine the growth of intracellular *Candida* cells, RAW264.7 cells were lysed with 0.1% Triton after incubation for an additional 5 h. After resuspension, serial dilution, and plating onto YPD plates, the phagocytized fungal cells were counted. The results are represented as means ± SD from three independent experiments.

### Adhesion of *C. glabrata* to human epithelial cells

Adhesion assays were performed by following a modified protocol described previously (59) and by using the human epithelial cell line A549. A549 cells were cultured in DMEM medium supplemented with 10% fetal bovine serum (FBS) at 37°C in 5% CO_2_. For the adhesion assay, *C. glabrata* cells suspended in DMEM medium containing 10% FBS were added to the 24-well plate where A549 cell monolayers had been prepared, at a MOI of 1:4 (macrophage: *Candida*). After incubation at 37°C for 4 h, nonadherent *C. glabrata* cells were carefully removed by washing with PBS. Epithelial cells and adherent *C. glabrata* cells were visualized by Giemsa staining.

### Murine model of *C. glabrata* infection

BALB/c and c57 mice were purchased from Beijing Vital River Laboratory Animal Technology Company. Mice were housed in a temperature-constant animal room (22°C) with a reversed dark/light cycle (7:00 a.m. on and 7:00 p.m. off) and 40%–70% humidity.

Neutropenia was induced in 19–21 g male BALB/c mice by administering cyclophosphamide intraperitoneally (150 mg/kg of body weight per day) 3 days before challenge with *C. glabrata* infection and on the day of infection. For the infection, *C. glabrata* cells were delivered into the immunosuppressed mice through a tail vein injection at a dosage of 5 × 10^7^ cells. On day 4 post-infection, the infected kidneys, spleens, and livers from these mice were harvested, homogenized, and cultured on agar plates supplemented with streptomycin (100 µg/mL) and ampicillin (50 µg/mL). Fungal colony forming units (CFUs) were counted after incubation at 30°C.

The DSS (Dextran Sulfate Sodium)-induced colitis experiment was conducted on 8- to 10-week-old female c57 mice, following previously described methods with some modifications (18, 33). To induce intestinal inflammation, the mice were given 2% DSS (36–50 kDa; MP Biomedicals) in their drinking water for a period of two weeks (from day 1 to day 14). On day 1, mice were inoculated with 10^8^ *C. glabrata* cells via oral gavage. After 14 days, the mice were euthanized, and their stomachs and colons were collected. These gastrointestinal (GI) tract segments were longitudinally dissected, and the intestinal contents were removed. Subsequently, the tissue samples were thoroughly washed in PBS to minimize contamination from *C. glabrata* within the lumen. The tissue homogenates were serially diluted and plated on agar plates containing streptomycin (100 µg/mL) and ampicillin (50 µg/mL). Fungal colonies were counted, and the results were expressed as *C. glabrata* CFU per gram of tissue.

### Quantitative PCR analysis

Total RNA was extracted and purified from *C. glabrata* cells incubated under the *in vitro* conditions using the RNeasy Mini kit. To remove DNA contamination, the RNA samples were treated with RNase-free DNase Set (Qiagen) for 15 min at room temperature. To determine the expression of *C. glabrata* genes during invasive infection, 19–21 g male BABL/c mice were inoculated with 7 × 10^8^ live *C. glabrata* cells by tail vein injection, and infected organs were taken at 24 h post-infection. Total RNA of the infected tissues of mice was extracted using the RNAprep Pure Tissue Kit (Tiangen). cDNA was synthesized using the Maxima H Minus cDNA Synthesis Master Mix with dsDNase (Thermo). For qRT-PCR analysis, iQ SYBR Green Supermix (Bio-Rad) was used in 96-well plates. The primers for qRT-PCR are listed in Table S1. Signals obtained from *ACT1* mRNA were used for normalization. All data are shown as the means of three independent experiments, with error bars representing the SD.

### Antifungal susceptibility testing

Susceptibility to fluconazole or caspofungin was assayed in a total volume of 0.1 mL/well with various concentrations of each drug in a liquid YPD medium. The 96-well plates were incubated in the dark at 30°C for 24 h before the optical density at 600 nm (OD_600_) was determined using a spectrophotometer (BioTek Instruments). Data were displayed as heat maps. Caspofungin (Selleck) was dissolved in DMSO. Fluconazole (Selleck) was dissolved in ethanol.

### Statistical and reproducibility

All experiments were performed with at least three biological repeats. No data were excluded from analyses. Analyses were conducted using GraphPad Prism. The results are expressed as the mean ± standard deviation (SD) except as indicated in the figure legends. All data were analyzed using unpaired Student’s *t*-tests. *P* values of less than 0.05 were considered statistically significant. Sample allocation was random in all experiments. No blinding was performed because none of the analyses reported involved procedures that could be influenced by investigator bias.

## Data Availability

RNA-Seq data that support the findings of this study have been deposited into GEO under the accession code GSE279281.

## SUPPLEMENTAL MATERIAL

Supplemental material msphere.00715-24-s0001.pdf Figures S1 to S5; Table S2.

Table S1 msphere.00715-24-s0002.xlsx *In vivo* RNA-seq data.

ASM does not own the copyrights to Supplemental Material that may be linked to, or accessed through, an article. The authors have granted ASM a non-exclusive, world-wide license to publish the Supplemental Material files. Please contact the corresponding author directly for reuse.
