# Supplementary material for: In vivo RNA sequencing reveals a crucial role of Fus3-Kss1 MAPK pathway in Candida glabrata pathogenicity
Source: mSphere. 2024 Oct 30;9(11):e00715-24. doi: 10.1128/msphere.00715-24 (PMC11580445; doi:10.1128/msphere.00715-24)
Supplement: Supplemental material — Figures S1 to S5; Table S2. [file msphere.00715-24-s0001.pdf]

A

```
                                                  *        20         *        40         *        60         *        80
C. glabrata    CAGL0J04290g  MGKKIVFNISSDFQLKSLLGEGAYGVVCSAVHKPTGEIVAIKKIVPFDKPLFALRTLREIKILRRFQHENIISIFDIQKP
S. cerevisiae  Fus3          MPKRIVYNISSDFQLKSLLGEGAYGVVCSATHKPTGEIVAIKKIEPFDKPLFALRTLREIKILKHFKHENIITIFNIQRP

                                      *       100         *       120         *       140         *       160
               CAGL0J04290g  ESFEKFNEVYIIQELMQTDLHRVIATQTLTDDHIQYFIYQTLRAIKTLHGSNVIHRDLKPSNILINSNCDLKVCDFGLAR
               Fus3          DSFENFNEVYIIQELMQTDLHRVISTQMLSDDHIQYFIYQTLRAVKVLHGSNVIHRDLKPSNLLINSNCDLKVCDFGLAR

                                      *       180         *       200         *       220         *       240
               CAGL0J04290g  IIDS--GENGDNEVDKIGMTEYVATRWYRAPEVMLTNAKYSRAMDIWSCGCILGELFLKRPLFPGKDYRHQLLLIFGLIG
               Fus3          IIDESAADNSEPTGQQSGMTEYVATRWYRAPEVMLTSAKYSRAMDVWSCGCILAELFLRRPIFPGRDYRHQLLLIFGIIG

                                      *       260         *       280         *       300         *       320
               CAGL0J04290g  TPDSSDKSMECIESRRARQYIASLQKYPPVRPLQKAFPGVNPVGVDLLSRMLVFDPRERISADDALAHPYLSTYHVPEDE
               Fus3          TPHS-DNDLRCIESPRAREYIKSLPMYP-AAPLEKMFPRVNPKGIDLLQRMLVFDPAKRITAKEALEHPYLQTYHDPNDE

                                      *       340         *       360
               CAGL0J04290g  PAGEPIPPDFFQFDQYKEVLTTKDLKKLLWNEIFHEQAT*
               Fus3          PEGEPIPPSFFEFDHYKEALTTKDLKKLIWNEIFS*----
```

B

```
                                      *        20         *        40         *        60         *        80
C. glabrata    CAGL0K04169g  MPRTITFNIPKEYKLVDLVGEGSYGIVCSATHLPTGNKVAIKRIRFPDSPPSDDGQGVPRLEEDDEHDNIAKVALIRTIR
S. cerevisiae  Kss1          MARTITFDIPSQYKLVDLIGEGAYGTVCSAIHKPSGIKVAIKKI----QPFS-----------------KKLFVTRTIR

                                      *       100         *       120         *       140         *       160
               CAGL0K04169g  EIKILSHFNNHENIITIFEKIKPMQSINFKEIYLVQELMETDLSRILASSN----TLTVDHIQYFLYQILRALKAIHSAK
               Kss1          EIKLLRYFHEHENIISILDKVRPVSIDKLNAVYLVEELMETDLQKVINNQNSGFSTLSDDHVQYFTYQILRALKSIHSAQ

                                      *       180         *       200         *       220         *       240
               CAGL0K04169g  VIHRDLKPSNILLNSNCDLKICDFGLARTYDPDDDASTMNGNNVGFLTEYVATRWYRAPEIMLNFQDYSTAIDIWSCGCV
               Kss1          VIHRDIKPSNLLLNSNCDLKVCDFGLARCLASSSDS---RETLVGFMTEYVATRWYRAPEIMLTFQEYTTAMDIWSCGCI

                                      *       260         *       280         *       300         *       320
               CAGL0K04169g  LAEMLFRKPIFPGKDYHHQLLLILDTLGTPSPEDIEAINYGRAKEYILSLPHFPKKDWSQLLGTD--NEMLLDILDKLMI
               Kss1          LAEMVSGKPLFPGRDYHHQLWLILEVLGTPSFEDFNQIKSKRAKEYIANLPMRPPLPWETVWSKTDLNPDMIDLLDKMLQ

                                      *       340         *       360         *       380         *       400
               CAGL0K04169g  FNPKRRITAEQALEHPFLATYHDPKDEPNFPPLNFKGDEFWQLDNCMETSNGYGTDDSNNLRANVTRRKLREILYQEMVT
               Kss1          FNPDKRISAAEALRHPYLAMYHDPSDEPEYPPLNLD-DEFWKLDNKIMRPE---------EEEEVPIEMLKDMLYDELMK

               CAGL0K04169g  PAPR*
               Kss1          TME*-
```

FIG S1 Alignment of *S. cerevisiae* MAPKs Fus3 and Kss1, and their potential homologs in *C. glabrata*. (A) Sequence alignment of *C. glabrata* CAGL0J04290g and *S. cerevisiae* Fus3. (B) Sequence alignment of *C. glabrata* CAGL0K04169g and *S. cerevisiae* Kss1. The single-letter code for amino acids is used. Consensus residues are shown with blue shade.

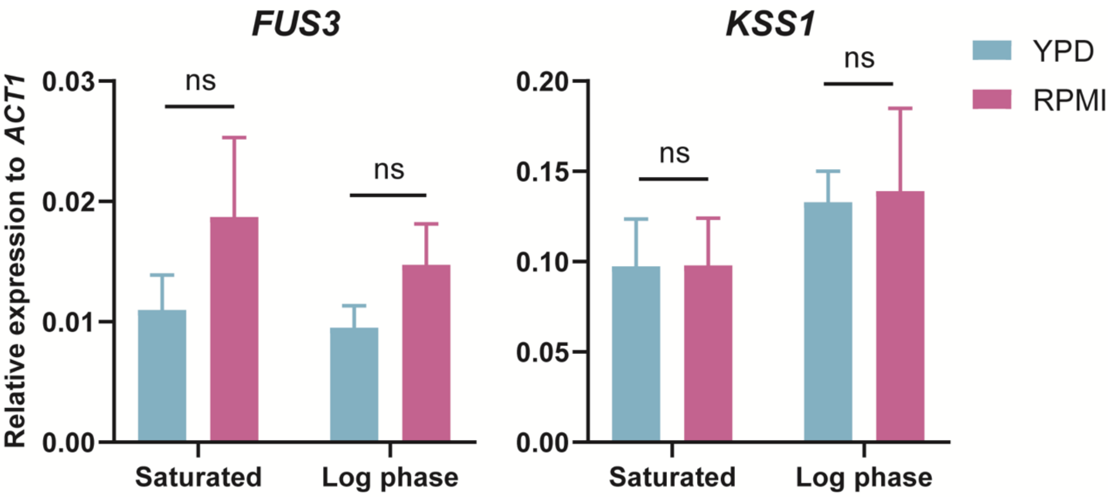

FIG S2 qRT-PCR analysis of *FUS3* and *KSS1* expression in wild type *C. glabrata* (CBS138) of indicated growth state in YPD or RPMI medium at 30°C. Mean data ± SD from three independent experiments was plotted. Significance was measured with an unpaired *t* test in GraphPad Prism. ns, no significance.

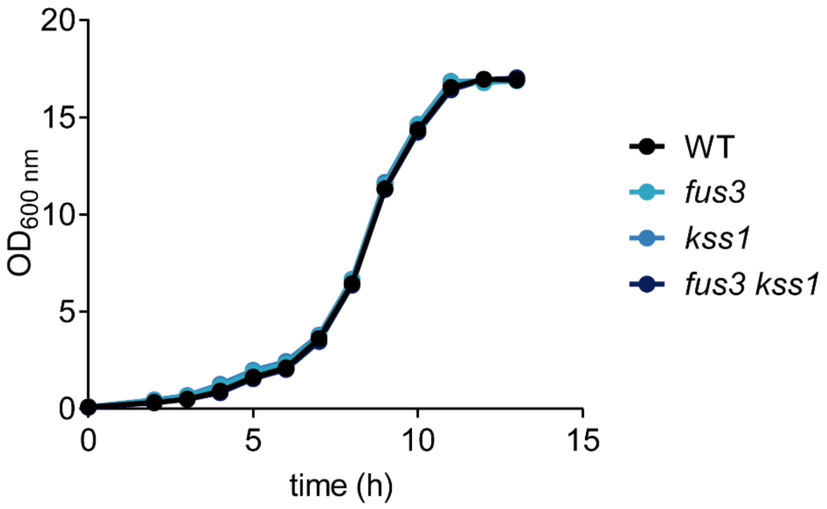

FIG S3 Growth assays for cells of wild type, single mutants *fus3* and *kss1*, and double mutant *fus3 kss1* incubated at 30°C in liquid YPD medium. *C. glabrata* cells from overnight cultures were diluted into YPD medium to OD_{600} 0.1 and then tested for growth at 30°C. Data are derived from one representative experiment. Three independent experiments yielding similar results were performed.

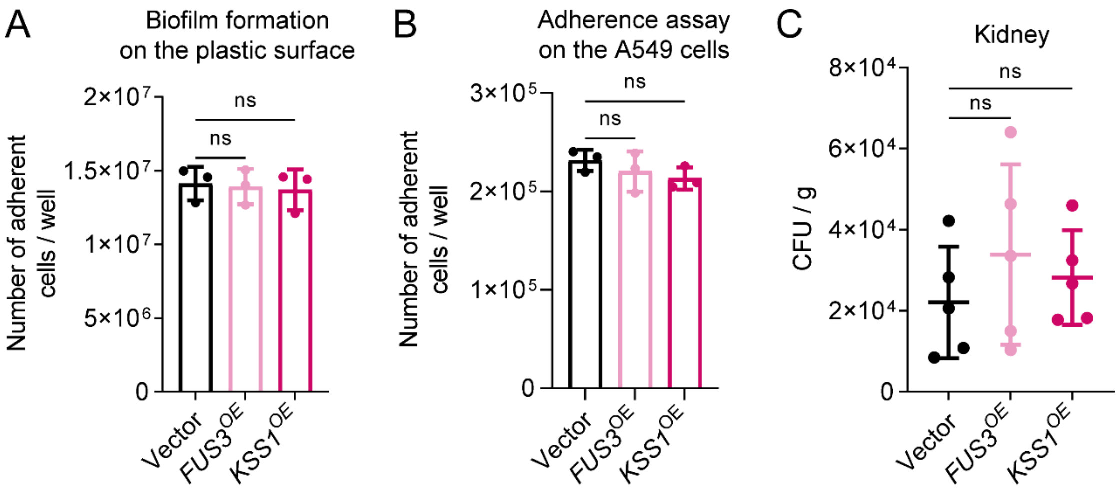

FIG S4 Functional analysis for the overexpression of MAPKs *FUS3* or *KSS1* in *C. glabrata*. (A) Cells of WT strain carrying *FUS3* or *KSS1* under the *TEF1* promoter or vector alone were inoculated onto 48-well plates with 500 µl SDB medium at 37°C for 48 h. The formation of biofilm was analyzed by the number of adherent cells. Values are the means ± SD from three independent experiments. (B) Adhesion assay on epithelial cell monolayers. *C. glabrata* cells of strains from (A) were inoculated with A549 cells in DMEM medium supplemented with 10% serum. After incubation for 4 h, non-adherent *Candida* cells were removed by washing with PBS. The numbers of adherent *Candida* cells are represented as means ± SD from three independent experiments. (C) The fungal loads of *C. glabrata* strains from (A) in kidneys of immunosuppressed mice were determined as in Fig. 2B. *n* = 5 mice. (A-C) Significance was measured with an unpaired *t* test in GraphPad Prism. ns, no significance.

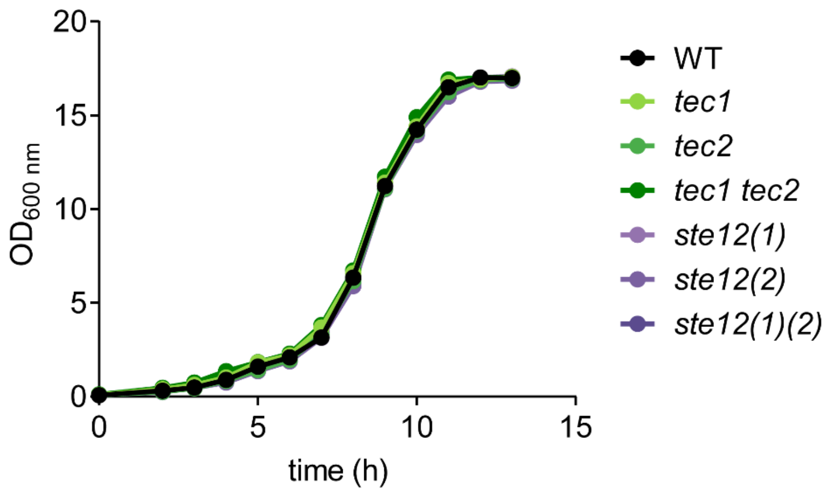

FIG S5 Growth assays for cells of wild type and indicated mutant strains incubated at 30°C in liquid YPD medium. The growth of *C. glabrata* cells was tested as in Fig. S2. Data are derived from one representative experiment. Three independent experiments yielding similar results were performed.

TABLE S2 Primers used in this study

	Sequence (5'-3')[a]	Purpose and features
1	GATCGAAGTTTTGAGAAATTTAACG	Guide *FUS3*
2	AAAACCGTTAAATTTCTCAAAACTTC	
3	TATAATTTCAATCTTTGATATACAGAAACCAGAAAGTTAAGAGAATTCTAACTAAGTCT	*FUS3* repair template
4	GTAAATCAGTTTGCATTAACTCTTGAATAATGTAGACTTAGTTAGAATTCTCTTAACTT	
5	CACCATTATACCGCGACC	*FUS3* knockout verification
6	GCGTCATCAGCTGATATTC	
7	GATCGATCGTCAAATACTTTGACTGG	Guide *KSS1*
8	AAAACCAGTCAAAGTATTTGACGATC	
9	GGAGTTAATGGAAACAGATCTGTCGAGAATTTTAGCATAGTCGAATTCTTAGCTGTAG	*KSS1* repair template
10	GAAGTATCTGGTATAAGAAATATTGAATATGATCCTACAGCTAAGAATTCGACTATG	
11	CGAGACAGCTTCCAGGTC	*KSS1* knockout verification
12	GTACACAACCACATGACC	
13	GATCGGATTTGCGGAACTTGAAGTGG	Guide *STE12(1)*
14	AAAACCACTTCAAGTTCCGCAAATCC	
15	GAAAGGAAGAAGTTCGAAGAGGGACTTTTCTCTGATTAGCGGAACTAGAATTCTTGAC	*STE12(1)* repair template
16	CTTTGATTTCGGCTGTTCTAAAGTTGCATCCAGTCAAGAATTCTAGTTCCGCTAATC	
17	CCGGTAGTCTATTTCCAC	*STE12(1)* knockout verification
18	GCTGGTTCTTGAATTGCG	
19	GATCGACTGGAAACCCTAACGACCTG	Guide *STE12(2)*
20	AAAACAGGTCGTTAGGGTTTCCAGTC	
21	ATGTCAATAGAGCTCCAAATTCTAATCAACGTGTTACTTGAATTCCTTAGGACTAGTGA	*STE12(2)* repair template
22	GTTTCAACTGTGTCTTGCGATGAAGAAAGATTAACTCACTAGTCCTAAGGAATTCAAGT	
23	CTACATCACAGGCACAG	*STE12(2)* knockout verification
24	CACTGCTGCTACTGCTT	
25	GATCGGTCAGCAGCTCAAATAGCACG	Guide *TEC1*
26	AAAACGTGCTATTTGAGCTGCTGACC	
27	GGTACAATATGAATAACTCTACCAGCACAAGTAATGTCTGATGATAAAATAGAATTCG	*TEC1* repair template
28	CGGTGGTCTGTGTGTGGCCGCTGCTGTTGCTGCTGCGAATTCTATTTTATCATCAGAC	
29	CTACACTTCTTCAAAGCC	*TEC1* knockout verification
30	CTGTGTTAGACATGGGTAC	
31	GATCGATTGGGAATTGTTATCAGGTG	Guide *TEC2*

32	AAAACACCTGATAACAATTCCCAATC	
33	GACTATTATGGAGGGAATTAGATTAACGTCATACTAACCTGATTAGAATTCCTAATGAC	*TEC2* repair template
34	GCTGCATTCTTATTTGTGGCCCAGAATTGTGCTGTCATTAGGAATTCTAATCAGGTTAG	
35	CGTGATTCGTTGTTAGATTC	*TEC2* knockout verification
36	CAGACGTAATGCGTCTTC	
37	TAAGTTTTCTAGAACTAGCGCGGCCGCATGGGCAAGAAAATTGTCTTC	*FUS3* overexpression
38	GAATTGTTAATTAAAGATCTCCGCGGTTAAGTTGCTTGTTCATGAAAG	
39	ATAAGAATGCGGCCGCGCCTACTGCCATAATCATTCCC	*KSS1* overexpression
40	TCCCCGCGGTCACCTAGGCGCTGGAGT	
41	ATGGATTCTGAAGTTGCTGC	*ACT1* qPCR
42	TGATACCTTGGTGTCTTGGT	
43	GAGTCGAGAAGAGCGAGAC	*FUS3* qPCR
44	GGATGTGCCAAAGCGTC	
45	CTTGTGGACCTTGTTGGTGA	*KSS1* qPCR
46	TGATCAACGCGACTTTGGC	
47	GTCGACCAGATCTCTACTGC	*SIT1* qPCR
48	CCGTAACCGTAACCACAGAC	
49	GTCCATCGGTGAATGGTATG	*FET3* qPCR
50	CCACCGACATTGACGATTCT	
51	GGGTATTCCAATGTTGCG	*FTR1* qPCR
52	CGACAGCTTCCAAACCTT	
53	GGCCAAGAACGTTGCTGATC	*YPS1* qPCR
54	GGTGGCGTACCGACTTC	

[a] Restriction sites are underlined.
